# The Motivational Make-Up of Workaholism and Work Engagement: A Longitudinal Study on Need Satisfaction, Motivation, and Heavy Work Investment

**DOI:** 10.3389/fpsyg.2020.01419

**Published:** 2020-06-30

**Authors:** Toon W. Taris, Ilona van Beek, Wilmar B. Schaufeli

**Affiliations:** ^1^Department of Social, Health and Organizational Psychology, Utrecht University, Utrecht, Netherlands; ^2^Dutch Police Academy, Apeldoorn, Netherlands

**Keywords:** workaholism, work engagement, need satisfaction, motivation, longitudinal research

## Abstract

Drawing on Ryan and Deci’s Self-Determination Theory, this study examines longitudinally how need satisfaction at work affects four forms of intrinsic and extrinsic work motivation and two types of heavy work investment (workaholism and work engagement). Using two-wave data from 314 Dutch employees, structural equation modeling supported our expectations that high need satisfaction was longitudinally associated with low levels of external and introjected regulation, and high levels of identified regulation and intrinsic motivation. Interestingly, none of these forms of regulation predicted later levels of work engagement and workaholism. Rather, high levels of work engagement predicted later high levels of intrinsic motivation and identified regulation, and high levels of workaholism predicted later low levels of intrinsic motivation and high levels of introjected regulation. Although this study did not support the expected longitudinal effects of motivation on the two types of heavy work investment examined in this study, it (a) underlined the important role of need satisfaction for motivation, (b) challenged previous ideas on the effects of motivation on workaholism and work engagement, and (c) revealed the different motivational correlates of work engagement and workaholism.

## Introduction

A large body of research has addressed the conceptualization, antecedents, consequences and outcomes of workaholism and work engagement (Quinones and Griffiths, 2015; Knight et al., 2017; for overviews). Moreover, the differences between these two forms of heavy work investment have frequently been studied (Harpaz and Snir, 2015; Shimazu et al., 2015a). For instance, drawing on Deci and Ryan (2000) self-determination theory (SDT), Van Beek et al. (2012) showed that (a) workaholic employees work hard in order to preserve and enhance feelings of self-worth and self-esteem, and because they personally value the associated outcomes, and (b) engaged employees work hard because they tend to experience their work activities as interesting, enjoyable, and satisfying.

Building on these and other findings, the present study extends current insights on the antecedents and correlates of heavy work investment in two respects. First, this study is among the first to examine how need satisfaction (a central concept in SDT; Deci et al., 2017; Van den Broeck and Van Beek, 2019) affects work motivation and how work motivation affects workaholism and work engagement across time. Although previous research has shown cross-sectionally that workaholics and engaged workers tend to differ in their underlying motivational regulation (Van Beek et al., 2012) longitudinal research on this issue is largely absent. By systematically comparing various models for the longitudinal associations between heavy work investment, need satisfaction and work motivation we aim to extend our understanding of the nature of heavy work investment: what drives workaholics and engaged workers? Second, we test the assumption that motivation mediates the associations between various types of need satisfaction on the one hand and work engagement and workaholism on the other. In this vein we aim to uncover the motivational processes that underlie the two different kinds of heavy work investment. By addressing these issues, we aim to enhance our understanding of the motivational correlates of different types of heavy work investment, which could help in developing effective strategies for enhancing work engagement and reducing workaholism.

### Two Kinds of Heavy Work Investment

Snir and Harpaz (2015) consider time and effort investments in work as the two core aspects of heavy work investment, representing its frequency and intensity, respectively. Moreover, they considered workaholism and work engagement as two different types of heavy work investment. Workaholism refers to “the tendency to work excessively hard and being obsessed with work, which manifests itself in working compulsively” (Schaufeli et al., 2009, p. 322), meaning that workaholic employees are chronically aroused and preoccupied with work. Consequently, they have little time for their spouses, family and friends, or for leisure activities (Shimazu et al., 2019) and do not experience the enjoyment and fulfillment accompanying such relationships or activities (Lyubomirsky and Nolen-Hoeksema, 1993). Frequent and/or continuous exposure to work without sufficient possibilities to recover may deplete workaholics’ energy resources as time goes by, possibly leading to burn-out (Gillet et al., 2017). Since workaholism is also linked to other adverse outcomes, such as job dissatisfaction (Burke and MacDermid, 1999) high turnover intention (Van Beek et al., 2014) and low work performance and high health complaints (Shimazu et al., 2015b) it can be considered a “bad” type of heavy work investment.

Conversely, work engagement is a positive work-related state of mind that is characterized by vigor (defined as “high levels of energy and mental resilience while working, the willingness to invest effort in one’s work, and persistence even in the face of difficulties”), dedication (“being strongly involved in one’s work, and experiencing a sense of significance, enthusiasm, inspiration, pride, and challenge”), and absorption (“being fully concentrated and happily engrossed in one’s work, whereby time passes quickly and one has difficulties with detaching oneself from work”) (Bakker et al., 2008, p. 188). Engaged employees work hard and derive great pleasure from it: they experience their work as interesting, enjoyable, and satisfying (Van Beek et al., 2011). Despite their high investments in their work, engaged employees participate in social activities, hobbies, and volunteer work (Burke, 2000; Bakker et al., 2008) resulting in sufficient possibilities for recovery (Van Beek et al., 2011). Furthermore, engaged employees perform well at work (Shimazu et al., 2019). Work engagement also relates to other beneficial outcomes such as high job satisfaction and organizational commitment (Knight et al., 2017) and good mental and physical health (Seppälä et al., 2012). Hence, work engagement is a “good” type of heavy work investment.

Since workaholism and work engagement are associated with adverse and beneficial outcomes, respectively, it is desirable to develop effective strategies for reducing workaholism and enhancing work engagement. Therefore, it is important to advance our knowledge of the *why* of workaholic and engaged employees’ behavior, that is, their motivation.

### Self-Determination Theory

Self-determination theory (SDT; Deci and Ryan, 2000) assumes that individuals are active, growth-oriented organisms, and that this growth-oriented tendency is fostered by fulfillment of three basic psychological needs: for autonomy, competence, and relatedness, respectively, (Deci et al., 2017). Need for autonomy refers to the need for experiencing freedom of choice and freedom to initiate behavior (Deci and Ryan, 2000). Need for competence refers to the need for completing challenging tasks successfully and achieving desired outcomes (White, 1959). Lastly, need for relatedness refers to the need for experiencing positive relationships with others and mutual respect (Baumeister and Leary, 1995). Although the association between need satisfaction and other concepts varies to a certain extent as a function of the type of need considered (Van den Broeck et al., 2016) satisfaction of these three needs tends to co-occur in a natural environment (Sheldon and Niemiec, 2006). Therefore, the present study focuses on the associations among need satisfaction in general (emphasizing what these three types of need satisfaction have in common), rather than on their possibly differential relationships with other concepts.

SDT posits that motivation, optimal functioning, and psychological well-being are affected by the extent to which environmental conditions allow satisfaction of the three needs and individuals can find or create the conditions necessary to satisfy these needs (Deci et al., 2017). The extent to which the three needs are satisfied explains how individuals orient themselves toward their social environment, their behavior, and what motivates them. As regards motivation, SDT makes a main distinction between intrinsic and extrinsic types of motivation (Deci et al., 2017). *Intrinsic motivation* refers to performing an activity because it is experienced as inherently enjoyable, interesting, and challenging. These activities are self-determined, i.e., they are conducted with a full sense of volition and choice. Conversely, *extrinsic motivation* refers to performing an activity because of its instrumental value, that is, extrinsically motivated individuals engage in an activity to obtain a desired outcome. SDT distinguishes among four types of extrinsic motivation that vary in the extent to which they are self-determined: external regulation, introjected regulation, identified regulation, and integrated regulation (Ryan and Deci, 2000). These types of extrinsic motivation are influenced by the degree to which the three innate psychological needs are fulfilled (Deci et al., 2017). The more these needs are satisfied, the more external social standards are transformed into personally endorsed values (internalization process), and the more self-determined the corresponding behaviors are.

*Externally regulated behavior* is motivated by external contingencies involving threats of punishments, or material and social rewards. This type of extrinsic motivation is experienced as fully controlling because individuals are regulated by contingent consequences that are administered by others and no internalization of external standards took place. Behavior that is governed by *introjected regulation* results from a partial internalization process in which individuals adopted external standards of self-worth and social approval, but without identifying with them. Individuals whose behavior is motivated by introjected regulation buttress themselves with feelings of self-worth and self-esteem when they manage to meet the adopted external standards, but they feel ashamed, guilty, and unworthy when they fail to do so (Ryan and Deci, 2002). Since introjected regulations are only partially internalized, individuals may experience a conflict between the adopted external standards and what they personally prefer (Ryan and Deci, 2000). Therefore, introjected regulation is experienced as somewhat controlling.

Behavior is motivated by *identified regulation* when individuals identify themselves with the underlying value of a behavior. For example, an employee whose behavior is motivated by identified regulation might be aware of the importance of it for his chosen career path. By recognizing the underlying value of a specific behavior, this regulation is more internalized than introjected regulation. Consequently, individuals experience some ownership of their behavior. Therefore, identified regulation is considered as somewhat autonomous behavior.

Lastly, behavior that is motivated by *integrated regulation* results from a full internalization process. Individuals identify themselves with the reasons for a particular behavior and have integrated these identifications with other aspects of the self. Like intrinsically motivated individuals, they experience their behavior as authentic and, thus, as self-determined. Since integrated regulation strongly resembles intrinsic motivation (Ryan and Deci, 2000) and because at present no instrument reliably measures this type of motivation (Gagné et al., 2015, p. 15–16), integrated regulation was not further examined here.

### The Present Study

Building on SDT, this study longitudinally examines the intrapersonal processes underlying workaholism and work engagement. Using a two-wave design with a 6-month time lag, four sets of hypotheses are simultaneously examined.

#### Need Satisfaction and Motivation

As noted previously, frustration of the needs for autonomy, competence, and relatedness undermines optimal motivation. Individuals with unfulfilled needs may search for autonomy, may work more to feel competent, or may search for company, and in the absence of satisfaction of these needs they will be motivated by external contingencies of punishment and reward (i.e., external regulation; Deci and Ryan, 2000; Deci and Vansteenkiste, 2004). Therefore, we expect that *need satisfaction has a negative effect on external regulation* (Hypothesis 1a).

Conversely, satisfaction of the needs for autonomy, competence and relatedness facilitates the transformation of external social standards into personally endorsed values. Specifically, individuals (partially) adopt a particular value because they feel connected with others who advocate that value (satisfaction of the need for relatedness) and because they feel competent with regard to behavior that represents that value (satisfaction of the need for competence), leading to introjected regulation (Vansteenkiste and Ryan, 2013). To foster fuller internalization of a value (and thus, identified regulation), individuals must also experience a sense of willingness and choice when conducting a behavior (satisfaction of the need for autonomy). Furthermore, satisfaction of these needs facilitates intrinsically motivated behaviors. Thus, we expect *positive effects of need satisfaction on introjected regulation* (Hypothesis 1b), *identified regulation* (Hypothesis 1c), *and intrinsic motivation* (Hypothesis 1d).

#### Motivation and Workaholism

Regarding motivation and heavy work investment, high levels of extrinsic motivation are likely to be positively associated with later workaholism, since workaholic employees work for its instrumental value (Van Beek et al., 2012). It has been suggested that workaholic employees have a negative self-image and lack self-confidence, leading to a high need to prove themselves at work in order to achieve a positive self-image (Mudrack, 2006; Robinson, 2014). Further, for workaholics disengagement from work causes distress and negative feelings, such as irritability, shame, and guilt (Killinger, 2006). This implies that for extrinsically motivated workers, putting high levels of effort (and many hours) in the job follows naturally from their motivations for working, helping them to increase or preserve self-esteem and self-worth (Ryan, 1982). These underlying motivations could also explain why workaholic employees experience a strong and uncontrollable inner drive to work hard. Furthermore, employees who find their work meaningful and important, and who identify themselves with their work goals (i.e., those who obtain relatively high scores on introjected and identified regualtion) are likely to put more effort in that job (i.e., to work harder and to feel more driven toward that job) than others (Van Beek et al., 2012). Since working hard and feeling driven toward the job are two key dimensions of workaholism, this implies that *introjected regulation and identified regulation will have a positive effect on workaholism* (Hypothesis 2a and Hypothesis 2b, respectively). A similar reasoning could apply to the possible association between external regulation and workaholism. However, previous cross-sectional research on this association (Van Beek et al., 2012) revealed that external regulation and workaholism were unrelated, possibly because workaholics are driven by an inner compulsion (i.e., internalized motivations) to work hard rather than just by external pressure (external regulation). Thus, no hypothesis is formulated for the possible association between external regulation and workaholism.

Intrinsically motivated workers work for the pleasure, enjoyment and interest that is inherent to their work activities. At first sight this could be a good reason to expect intrinsically motivated workers to show symptoms of workaholism. That is, intrinsically motivated workers could feel tempted to work long hours, which could ultimately lead to energy depletion and adverse consequences for health and well-being (cf. Nerstadt et al., 2019 who show that high levels of engagement – a concept closely related to intrinsic motivation – can increase the risk of burnout). Alternatively, it could be argued that intrinsically motivated workers lack the compulsive drive that is typical for workaholics (Schaufeli et al., 2009) suggesting that intrinsically motivated workers may well work hard, but – contrary to workaholics – their work will not feel like a compulsion and they will not put *excessive* effort in their jobs, since this will have negative consequences: there is little inherent pleasure in being exhausted and performing below par. This reasoning was confirmed by Van Beek et al. (2012), who found that high levels of workaholism were associated with low levels of intrinsic motivation. Given the limited evidence on this matter we tentatively propose that *intrinsic motivation will have a negative effect on workaholism* (Hypothesis 2c).

#### Motivation and Work Engagement

Engaging in work activities that one considers interesting, enjoyable and satisfying – i.e., in intrinsically motivating work – may lead workers to work for its own sake, and it is not difficult to understand why such workers tend to report relatively high levels of work engagement (Van Beek et al., 2012). Furthermore, engaged employees work because they value their work, suggesting that they identify themselves with their work goals. Since many jobs include interesting and enjoyable tasks as well as more mundane and unpleasant tasks, even engaged employees will also to some degree be extrinsically motivated (Van Beek et al., 2012). Prior studies have demonstrated that engaged employees believe in their capabilities to attain work goals and that good things will happen to them (Xanthopoulou et al., 2007). Individuals with such positive beliefs are likely to pursue self-concordant goals (Judge et al., 2005). By pursuing work goals that fit their ideals, interests, and values, these individuals are likely to act with a sense of volition and to actualize their growth-oriented nature (Deci and Vansteenkiste, 2004). As a result, they might experience a sense of energy while working, get strongly involved in their work, and have difficulties to detach from it. Hence, *intrinsic motivation and identified regulation will have a positive effect on work engagement* (Hypothesis 3a and Hypothesis 3b, respectively). One could argue that a similar reasoning could apply to the association between introjected regulation and work engagement, since introjected regulation also involves the internalization of external standards. However, since these standards are only partially internalized, workers could experience conflict between these adopted standards and their own preferences (Ryan and Deci, 2000) which is likely to weaken the association between introjected regulation and work engagement. Therefore, no particular hypothesis is formulated for this association.

**FIGURE 1:**
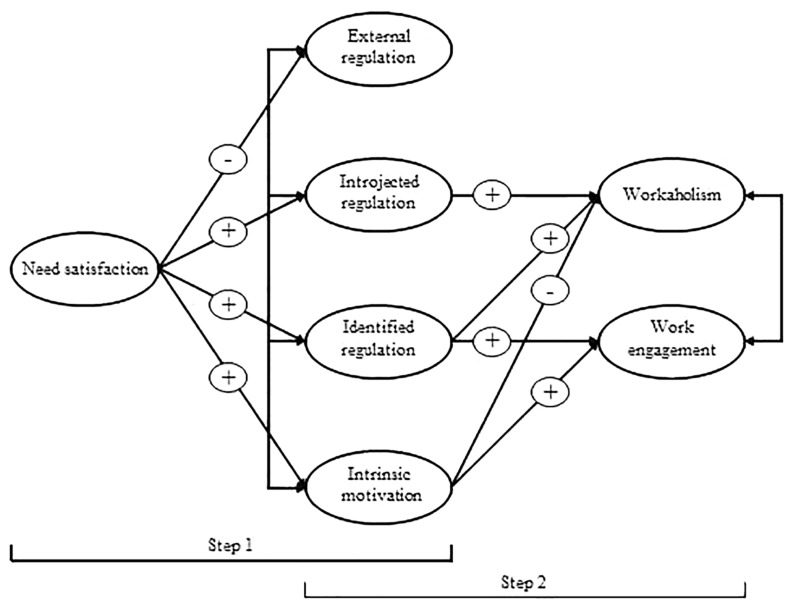
Model for the relationships among need satisfaction, regulatory styles, workaholism, and work engagement.

Figure 1 presents our research model. Basically, this model states that the associations between need satisfaction on the one hand and heavy work investment (workaholism and work engagement) are mediated by the four types of motivation included in this study (Hypothesis 4). The mediational hypotheses implied in the model presented in Figure 1 cannot properly be tested longitudinally, as this would require at least three waves of data. However, using the approach proposed by Cole and Maxwell (2003) two main conditions that should be met for mediation to occur can be tested longitudinally. That is, if mediation occurs, then need satisfaction should longitudinally predict motivation (step 1), and motivation should longitudinally predict engagement and workaholism (step 2). Hypothesis 4 is supported if Hypotheses 1a-d (on the effects of need satisfaction on motivation), Hypotheses 2a-c (the effects of motivation on work engagement) and Hypotheses 3a-b (motivation and workaholism) are at least partly supported. Note that we cannot properly test whether the associations between need satisfaction on the one hand and workaholism and work engagement on the other are fully or only partially mediated by the four regulation types, as this would require three waves of data. Thus, we present no hypotheses concerning full or partial mediation.

## Materials and Methods

### Sample and Procedure

Participants were recruited through a call on a Dutch internet site addressing career-related issues. Visitors of this site were invited to complete an online questionnaire concerning work motivation. The study was carried out in accordance with the ethical guidelines of the American Psychological Association and our local ethical review board. That is, although studies using standardized self-report surveys in which participants are not deceived and in which no intervention is implemented or evaluated are formally exempted from the approval of an institutional ethics committee, participants were a priori informed about the aims and design of the study. Moreover, before starting the questionnaire they were informed that participation was voluntary and anonymous. Participants did not receive any monetary compensation and could withdraw from the study whenever they wanted.

In total, 3,465 visitors responded to our call, 1,896 of which (55%) finished the questionnaire. Only complete records were included. No missing data occurred because respondents were required to fill in all questions. Of these 1,896 respondents, 113 respondents were unemployed and 10 respondents completed the questionnaire more than once or failed to do so seriously (i.e., they answered (nearly) all of its 54 items with “never”/”totally disagree”). Approximately 6 months later, 1,773 respondents who had indicated that they were willing to participate in the follow-up to this study and who had provided their email address, were invited by email to fill out the questionnaire for a second time.

In total, 330 respondents completed both questionnaires (18.6%). It is possible that the actual response rate was higher. For example, respondents might have retired or might have changed their email address after the first measurement. Of these 330 respondents, 281 respondents had retained their job, 33 respondents had changed their job, and 16 respondents had lost their jobs and were excluded from further analyses. Therefore, the present study included 314 participants (132 males with a mean age of 47.2 years, *SD* = 8.5, and 182 females with a mean age of 44.2 years, *SD* = 8.8).

To test for possibly selective drop-out of participants, respondents who filled out the questionnaire at Time 1 only (*N* = 1,459) were compared to respondents who filled out the questionnaire at both occasions (*N* = 314), i.e., our study participants. A Pearson chi-square test showed a more equitable male-to-female ratio in the latter group: 58% of our study participants was female, compared to 67.6% of the respondents who dropped out at Time 2, *χ*^2^(*df* = 1) = 10.80, *p* < 0.01. Furthermore, independent samples *t*-tests showed that our study participants were older (*M*_*age*_ = 45.47 years) and higher educated (*M*_*education*_ = 4.70) than the drop-outs (*M*_*age*_ = 41.92 years and *M*_*education*_ = 4.54), *t*(*df* = 500,83) = −6.32, *p* < 0.01 and *t*(*df* = 488,93) = −2.23, *p* < 0.05, respectively. The two groups did not differ in terms of years of job experience, *F*(5, 1767) = −0.933, *ns*. Finally, multivariate analysis of variance showed that the two groups did not differ on the study variables at Time 1, *F*(11, 1761) = 1.62, *ns*.

### Measures

*Workaholism* was measured with the Dutch Work Addiction Scale (DUWAS; Schaufeli et al., 2009) that consists of two subscales: Working excessively and working compulsively. *Working excessively* is measured with 9 items, including “I seem to be in a hurry and racing against the clock”, whereas *Working compulsively* is measured with seven items, such as “I feel that there’s something inside me that drives me to work hard” (1 = “(almost) never”, 4 = “(almost) always”). Since workaholism can be regarded as a syndrome (i.e., a combination of working excessively and working compulsively; Van Beek et al., 2011) a composite workaholism score was used in the present study.

*Work engagement* was measured with the short version of the Utrecht Work Engagement Scale (UWES; Schaufeli et al., 2006) that consists of three subscales: Vigor, dedication, and absorption. *Vigor* was measured with three items, including “At my work, I feel strong and vigorous”, *dedication* was measured with three items, such as “I am enthusiastic about my job”, and *absorption* was measured with three items as well, including “I am immersed in my work” (0 = “never”, 6 = “always”).

*Motivation* was measured with the Multidimensional Work Motivation Scale (MWMS; Gagné et al., 2015). Four subscales were used: External social regulation, introjected regulation, identified regulation, and intrinsic motivation. *External social regulation* was measured with three items, including “I work to get others’ approval (e.g., supervisor, colleagues, family, clients).” *Introjected regulation* was measured with four items, such as “I work because I must prove myself that I can.” *Identified regulation* was measured with three items, including “I work because I personally consider it important to put efforts in this job.” *Intrinsic motivation* was measured with three items, such as “I work because I have fun doing my job.” Items were scored on a 5-point scale (1 = “totally disagree”, 5 = “totally agree”).

*Need satisfaction* was measured with the Work-related Basic Need Satisfaction scale (W-BNS; Van den Broeck et al., 2010) that includes three subscales: autonomy satisfaction, competence satisfaction, and relatedness satisfaction. *Autonomy satisfaction* was measured with six items, including “I feel like I can be myself at my job,” *competence satisfaction* was measured with four items, such as “I really master my tasks at my job,” and *relatedness satisfaction* was measured with six items as well, including “At work, I feel part of a group” (1 = “totally disagree,” 5 = “totally agree”).

### Statistical Analyses

Structural Equation Modeling (SEM) methods as implemented in AMOS 21.0 (Arbuckle, 2012) were used to test the hypotheses. Maximum likelihood estimation was applied and the goodness-of-fit of the tested models was evaluated using the χ*^2^* test statistic, the Goodness-of-Fit Index (GFI), the Comparative Fit Index (CFI), the Normed Fit Index (NFI), the Tucker-Lewis Index (TLI) and the Root Mean Square Error of Approximation (RMSEA). Values larger than 0.90 for GFI, CFI, NFI, and TLI, and 0.10 or lower for RMSEA indicate reasonable model fit (Byrne, 2009; Hopwood and Donnellan, 2010).

#### Preliminary Analyses: Measurement Models

Before testing the research model presented in Figure 1, a series of confirmatory factor analyses was conducted for the measurement model employed in this study (cf. Table 1). For each study wave, four models were tested. The first was the *independence model* (a), assuming that all 11 concepts included in this study (workaholism; intrinsic, external social, introjected and identified regulation; vigor, dedication and absorption; and autonomy, competence and relatedness satisfaction) were statistically independent. The *single-factor model* (b) assumed that all 11 concepts were observed indicators of a single underlying dimension. Model (c) proposed that vigor, dedication and absorption were indicators of a latent concept representing “work engagement,” while autonomy, competence and relatedness satisfaction were considered to be indicators of a latent concept “need satisfaction.” This model assumes that all seven study concepts (i.e., the two latent dimensions and the other five study concepts) were unrelated. Finally, model (d) is identical to model (c), but assumes that all seven study concepts are related.

**TABLE 1:** Fit indices for the measurement models (*N* = 314).

Model	χ ^2^	*df*	GFI	CFI	NFI	TLI	RMSEA	Model comparisons	Δχ ^2^	Δ*df*
**Wave 1**										
(a): Independence model	1893.12	55	0.37	0.00	0.00	0.00	0.33			
(b): Single-factor model	475.56	44	0.75	0.77	0.75	0.71	0.18	(a) vs. (b)	1417.56***	11
(c) Proposed model, no covariances	1079.26	49	0.59	0.44	0.43	0.37	0.23	(a) vs. (c)	813.86***	6
(d) Proposed model, covariances allowed	151.72	28	0.93	0.93	0.92	0.87	0.12	(c) vs. (d)	1079.26***	27
**Wave 2**										
(a) Independence model	2038.16	55	0.22	0.00	0.00	0.00	0.34			
(b) Single-factor model	487.17	44	0.75	0.78	0.76	0.72	0.18	(a) vs. (b)	1550.99***	11
(c) Proposed model, no covariances	1106.16	49	0.59	0.47	0.46	0.40	0.26	(a) vs. (c)	932.00***	6
(d) Proposed model, covariances allowed	108.68	28	0.94	0.96	0.95	0.92	0.09	(c) vs. (d)	997.48***	27
**Measurement invariance tests: Longitudinal comparisons of model (*d*)**										
(e) Unconstrained model (baseline model)	321.33	122	0.92	0.96	0.94	0.93	0.07			
(f) Baseline model (e), plus factor loadings of engagement and need satisfaction constrained across time	327.80	128	0.92	0.96	0.94	0.93	0.07	(e) vs. (f)	6.47	6
(g) Model (f), plus error variances of the indicators of engagement and need satisfaction constrained across time^#^	337.89	134	0.91	0.96	0.94	0.93	0.07	(f) vs. (g)	10.09	6

*^#^The unstandardized loadings of the observed indicators vigor, dedication and absorption on the latent factor Work engagement were 1.08, 1.36, and 1.01, respectively, and their Time 1/Time 2 standardized loadings were 0.83 and 0.86 (vigor), 0.98 and 0.97 (dedication), and 0.80 and 0.83 (absorption). The unstandardized loadings of the observed indicators competence satisfaction, autonomy satisfaction and relatedness satisfaction on the latent factor Need satisfaction were 0.32, 0.42, and 0.63, respectively. Their Time 1/Time 2 standardized loadings were 0.50 and 0.54 (competence), 0.58 and 0.59 (autonomy) and 0.88 and 0.89 (relational). All loadings were significant at *p* < 0.001.*

Table 1 shows that models (a) and (c) – that assumed that the study variables were largely or wholly unrelated – did not fit the data at both study waves. Single-factor model (b) improves strongly on these two models, but its fit was far from acceptable for both study waves. Conversely, model (d), the model proposed for this study, fitted the data reasonably well across both study waves, with only RMSEA and TLI not meeting their cutoff values at Time 1. Subsequent measurement invariance tests indicated that model (d) accounted reasonably well for the data at both study waves. Specifically, model (e), testing whether the basic measurement model applied to both study waves, showed good fit (*χ*^2^ with 122 *df* = 321.33; GFI, CFI, NFI and TLI > 0.90; RMSEA = 0.07). Constraining the loadings of engagement and need satisfaction to be equal for both study waves (model (f)) did not lead to a deterioration of model fit, Δχ^2^ with 6 *df* = 6.47, *ns*. Neither did constraining the error variances of the indicators of engagement and need satisfaction to be equal across time (model (g)) lead to a significant decrease in model fit, Δχ^2^ with 6 *df* = 10.09, *ns*, while overall model fit was still acceptable (cf. Table 1). Thus, work engagement and need satisfaction were considered as latent variables, with their factor structure being invariant across both study waves.

#### Structural Analyses

Our research model (Figure 1) is a mediation model, but since we have only two waves of data available this model could not be tested in full using the present two-wave study design. Therefore, we used the two-step procedure recommended by Cole and Maxwell (2003) to obtain an approximation of the mediation process using two-wave data. Cole and Maxwell recommend two subsequent longitudinal tests: (1) investigating the causal (longitudinal) relationships between the predictor A (i.e., need satisfaction) and the mediator B (i.e., motivational regulations); and (2) investigating the causal (longitudinal) relationships between the mediator B and the outcome C (i.e., workaholism and work engagement). If predictor A longitudinally affects mediator B *and* if mediator B longitudinally affects outcome C, it is plausible that the association between A and C is mediated by B, and an indication of the magnitude of the mediation effect can be obtained by multiplying the longitudinal effect of A on B by the longitudinal effect of B on C (Cole and Maxwell, 2003). By examining the hypothesized relations in two steps, we also took into account the ratio of the number of participants to the number of free parameters (i.e., model complexity; Kline, 2005). First, we examined the longitudinal relations between need satisfaction and the different types of motivation (Step 1; see Figure 1). Second, we examined the longitudinal relations among the different types of motivation and the two types of heavy work investment in the present study: workaholism and work engagement (Step 2).

In both steps, four different models were compared using the delta chi-square test statistic (Δχ*^2^*): a stability model, a causality model, a reversed causality model, and a reciprocal model. In the *stability model*, each factor as measured at Time 1 predicted that same factor as measured at Time 2. For example, need satisfaction at Time 1 predicted need satisfaction at Time 2, external regulation at Time 1 predicted external regulation at Time 2, et cetera (step 1). In the *causality model*, the stability model was extended with cross-lagged paths between need satisfaction at Time 1 and the different types of motivation at Time 2 (step 1), and with cross-lagged paths between the different types of motivation at Time 1 and the two types of heavy work investment at Time 2 (step 2). In the *reversed causality model*, the stability model was extended with cross-lagged paths in the opposite direction, i.e., paths of motivation at Time 1 on need satisfaction at Time 2 (step 1), and from heavy work investment at Time 1 on motivation at Time 2 (step 2). Lastly, in the *reciprocal model*, the cross-lagged paths of the causality model *and* the reversed causality model were added to the stability model. The cross-lagged paths in the causality model, the reversed causality model, and the reciprocal model were relevant to the hypotheses. In all models, synchronous correlations were allowed among the latent/manifest variables at Time 1 and among the error terms of the latent/manifest variables at Time 2 (cf. Hakanen et al., 2008). In addition, following the recommendations of Pitts et al. (1996) correlations were allowed between the error terms of the indicator variables of the latent variables at Time 1 and the corresponding error terms of the indicator variables of the latent variables at Time 2.

## Results

Table 2 presents the means, standard deviations, correlations, and reliabilities (Cronbach’s alpha) for the study variables.

**TABLE 2:** Means, standard deviations, correlations, and Cronbach’s alpha coefficients on the diagonal (*N* = 314).

Variables		*M*	*SD*	1	2	3	4	5	6	7	8	9	10
1	Workaholism T1	2.03	0.53	0.74^a^									
2	Workaholism T2	2.00	0.51	0.73	0.73^a^								
3	Vigor T1	3.18	1.30	–0.11	–0.10	0.91							
4	Vigor T2	3.26	1.27	–0.04	–0.14	0.73	0.93						
5	Dedication T1	3.48	1.40	–0.02	–0.02	0.81	0.62	0.92					
6	Dedication T2	3.56	1.38	0.01	–0.08	0.61	0.83	0.70	0.93				
7	Absorption T1	3.10	1.26	0.14	0.13	0.73	0.60	0.79	0.62	0.85			
8	Absorption T2	3.08	1.22	0.09	0.03	0.57	0.78	0.57	0.80	0.70	0.85		
9	Satisfaction with autonomy T1	3.37	0.76	–0.29	–0.27	0.61	0.48	0.68	0.54	0.41	0.34	0.86	
10	Satisfaction with autonomy T2	3.42	0.79	–0.18	–0.35	0.49	0.59	0.52	0.68	0.38	0.49	0.72	0.87
11	Satisfaction with competence T1	4.01	0.64	–0.11	–0.06	0.34	0.25	0.22	0.20	0.23	0.18	0.21	0.17
12	Satisfaction with competence T2	4.10	0.58	–0.03	–0.12	0.33	0.36	0.25	0.31	0.29	0.35	0.23	0.28
13	Satisfaction with relatedness T1	3.39	0.72	–0.23	–0.22	0.47	0.45	0.47	0.40	0.31	0.29	0.50	0.42
14	Satisfaction with relatedness T2	3.40	0.73	–0.20	–0.26	0.39	0.53	0.38	0.50	0.29	0.39	0.45	0.59
15	External regulation T1	2.61	0.94	0.30	0.17	–0.13	–0.13	–0.12	–0.11	–0.01	–0.03	–0.23	–0.12
16	External regulation T2	2.50	0.90	0.17	0.23	–0.13	–0.15	–0.10	–0.11	–0.02	–0.05	–0.20	–0.22
17	Introjected regulation T1	2.85	0.85	0.40	0.32	–0.17	–0.11	–0.12	–0.06	–0.00	0.07	–0.24	–0.16
18	Introjected regulation T2	2.73	0.82	0.30	0.38	–0.19	–0.15	–0.10	–0.10	–0.04	–0.01	–0.24	–0.24
19	Identified regulation T1	3.96	0.74	0.10	0.13	0.38	0.30	0.41	0.33	0.40	0.31	0.34	0.30
20	Identified regulation T2	4.03	0.65	0.14	0.08	0.30	0.37	0.34	0.40	0.32	0.37	0.29	0.33
21	Intrinsic motivation T1	3.58	0.90	–0.09	–0.11	0.67	0.53	0.77	0.62	0.58	0.49	0.67	0.52
22	Intrinsic motivation T2	3.69	0.86	–0.10	–0.21	0.53	0.70	0.62	0.79	0.45	0.61	0.55	0.71
11	Satisfaction with competence T1	0.87											
12	Satisfaction with competence T2	0.56	0.84										
13	Satisfaction with relatedness T1	0.12	0.14	0.82									
14	Satisfaction with relatedness T2	0.07	0.14	0.72	0.86								
15	External regulation T1	–0.19	–0.21	–0.08	–0.03	0.78							
16	External regulation T2	–0.17	–0.29	–0.09	–0.11	0.49	0.77						
17	Introjected regulation T1	–0.30	–0.19	–0.20	–0.18	0.52	0.41	0.76					
18	Introjected regulation T2	–0.21	–0.24	–0.13	–0.16	0.36	0.59	0.55	0.75				
19	Identified regulation T1	0.19	0.19	0.34	0.18	–0.03	–0.08	0.02	0.03	0.86			
20	Identified regulation T2	0.10	0.19	0.27	0.23	–0.01	–0.09	0.12	0.04	0.58	0.80		
21	Intrinsic motivation T1	0.16	0.25	0.48	0.42	–0.10	–0.09	–0.10	–0.12	0.49	0.36	0.85	
22	Intrinsic motivation T2	0.10	0.24	0.39	0.55	–0.08	–0.14	–0.09	–0.12	0.29	0.40	0.64	0.86

*Correlations of 0.12 and over significant at *p* < 0.05; correlations of 0.15 and over significant at *p* < 0.01; ^*a*^correlation between the two subscales of the DUWAS: Working Excessively and Working Compulsively; α Working Excessively T1 = 0.81; α Working Compulsively T1 = 0.84; α Working Excessively T2 = 0.81; α Working Compulsively T2 = 0.84.*

### Testing the Research Models

#### Need Satisfaction and Motivation

Table 3 shows the fit indices for the study models. The analyses in the first step revealed that the reciprocal model (M1_*reciprocal*_) fitted the data well, χ^2^(*N* = 314, *df* = 49) = 126.54, GFI = 0.95, CFI = 0.96, NFI = 0.94, TLI = 0.93, RMSEA = 0.071, and significantly better than the stability model (M1_*stability*_), Δχ^2^(*N* = 314, *df* = 8) = 27.96, *p* < 0.01, and the reversed causality model (M1_*reversed causality*_),Δχ^2^(*N* = 314, *df* = 4) = 21.94, *p* < 0.01. The fit of the reciprocal model was comparable to that of the causality model (M1_*causality*_), Δχ^2^(*N* = 314, *df* = 4) = 3.99, *p* > 0.05. The causality model revealed that *all* paths from need satisfaction (Time 1) to modes of motivational regulation (Time 2) were significant, except for that of identified regulation (0.10; *p* = 0.07). Conversely, *no* paths from motivational regulation (Time 1) to need satisfaction (Time 2) were significant; for external, introjected, identified, and intrinsic motivational regulation these path coefficients were 0.08 (*p* = 0.09), −0.01 (*p* = 0.98), 0.02 (*p* = 0.58), and 0.07 (*p* = 0.54), respectively. The results of the reciprocal model agree completely with the combined results of the causality and reversed causal models; all path coefficients from need satisfaction (Time 1) to motivational regulation (Time 2) are significant (again, with the exception of the effect of T1 need satisfaction on T2 identified regulation), whereas all reversed causal paths were non-significant.

**TABLE 3:** Fit indices for the models (*N* = 314).

Model	χ ^2^	*df*	GFI	CFI	NFI	TLI	RMSEA	Model comparisons	Δχ ^2^	Δ *df*
**Step 1: testing the relations between need satisfaction and motivation**										
M1_*stability*_	154.50	57	0.94	0.95	0.93	0.92	0.074			
M1_*causality*_	130.53	53	0.94	0.96	0.94	0.93	0.068	M1_*stability*_-M1_*causality*_	23.97**	4
M1_*reversed*_	148.48	53	0.94	0.95	0.93	0.92	0.076	M1_*stability*_-M1_*reversed*_	6.02	4
M1_*reciprocal*_	126.54	49	0.95	0.96	0.94	0.93	0.071	M1_*stability*_-M1_*reciprocal*_	27.96**	8
M1_*final*_	133.65	54	0.94	0.96	0.94	0.93	0.069	M1_*causality*_-M1_*reciprocal*_	3.99	4
								M1_*reversed*_-M1_*reciprocal*_	21.94**	4
**Step 2: testing the relations between motivation and heavy work investment**										
M2_*stability*_	233.84	75	0.92	0.96	0.94	0.93	0.082			
M2_*causality*_	209.63	70	0.93	0.96	0.94	0.93	0.080	M2_*stability*_-M2_*causality*_	24.21**	5
M2_*reversed*_	176.27	70	0.94	0.97	0.95	0.95	0.070	M2_*stability*_-M2_*reversed*_	57.57**	5
M2_*reciprocal*_	167.75	65	0.94	0.97	0.95	0.95	0.071	M2_*stability*_-M2_*reciprocal*_	66.09**	10
M2_*final*_	179.97	71	0.94	0.97	0.95	0.95	0.070	M2_*causality*_-M2_*reciprocal*_	41.88**	5
								M2_*reversed*_-M2_*reciprocal*_	8.52	5

**FIGURE 2:**
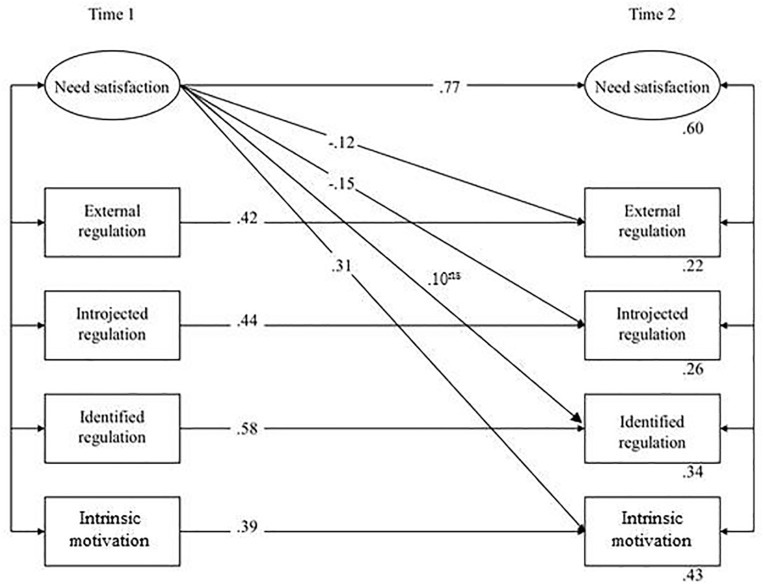
Final SEM model: relations between need satisfaction and motivation (the casuality model, model M1).

Since the causality model was more parsimonious and essentially contained the same information in terms of significant paths, the reciprocal model was rejected in favor of the causality model. Subsequently non-significant paths were removed from the causality model for three reasons (cf. McCoach, 2003). First, including them would lead to a relatively complicated, difficult-to-interpret and non-parsimonious model. Second, deleting non-significant effects increases the number of degrees of freedom for the model test and, thus, increases statistical power. Third, in structural equation modeling parameter estimates are dependent on the model that is estimated, i.e., omitting a particular parameter could potentially lead to changes in other parameters. Including non-significant parameter estimates may thus affect (and even bias) the other parameter estimates. This final, trimmed model (M1_*final*_) still fitted the data adequately, χ^2^(*N* = 314, *df* = 54) = 133.65, GFI = 0.94, CFI = 0.96, NFI = 0.94, TLI = 0.93, RMSEA = 0.069. Figure 2 presents the significant effects of need satisfaction on different types of motivation. For clarity, this figure does not include the correlations between the error terms of the indicator variables of need satisfaction as measured at Time 1 and Time 2 (Pitts et al., 1996).

Hypothesis 1a stated that need satisfaction would have a negative effect on external regulation. The findings presented in Figure 2 show that need satisfaction at Time 1 significantly influenced external regulation (β = −0.12) at Time 2 (Hypothesis 1a supported). Furthermore, Hypotheses 1b–1d proposed that need satisfaction would have a positive effect on introjected regulation, identified regulation, and intrinsic motivation, respectively. Need satisfaction at Time 1 influenced introjected regulation at Time 2 negatively rather than positively (β = −0.15, Hypotheses 1b rejected). Further, need satisfaction at Time 1 did not significantly predict identified regulation at Time 2 (Hypothesis 1c rejected). In line with our expectations, need satisfaction at Time 1 significantly predicted intrinsic motivation at Time 2 (β = 0.31; Hypothesis 1d supported).

#### Motivation and Heavy Work Investment

**FIGURE 3:**
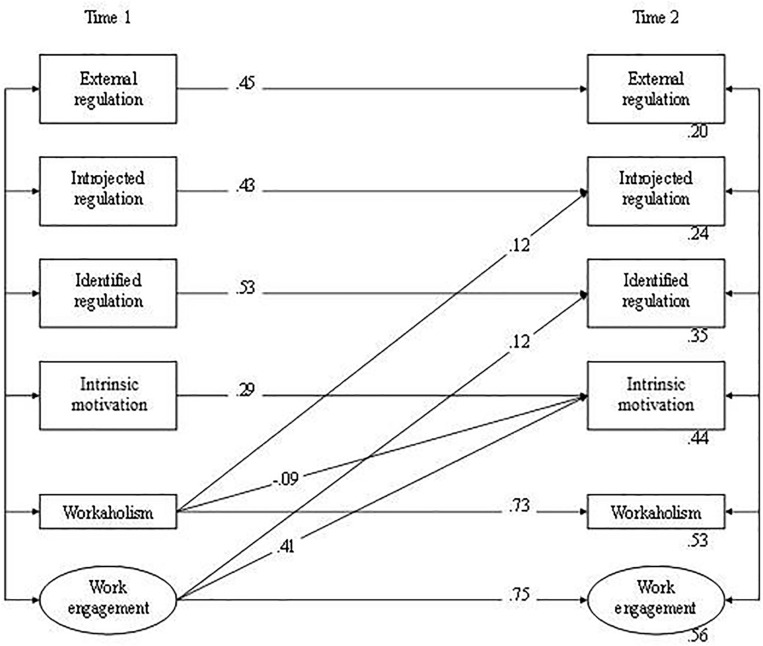
Final SEM model: relations between motivation and heavy work investment (the reversed casuality model, model M2).

The analyses in the second step showed that the reciprocal model (M2_*reciprocal*_) fitted the data well, χ^2^(*N* = 314, *df* = 65) = 167.75, GFI = 0.94, CFI = 0.97, NFI = 0.95, TLI = 0.95, RMSEA = 0.071, and significantly better than the stability model (M2_*stability*_), Δχ^2^(*N* = 314, *df* = 10) = 66.09, *p* < 0.01, and the causality model (M2_*causality*_), Δχ^2^(*N* = 314, *df* = 5) = 41.88, *p* < 0.01. The causality model revealed that only the path coefficients from identified regulation to workaholism (0.10, *p* = 0.02) and from intrinsic motivation to work engagement (0.24, *p* < 0.001) were significant. The remaining paths from introjected regulation and intrinsic motivation to workaholism (0.01, *p* = −0.86 and −0.07, *p* = 0.09, respectively), as well as the path from identified regulation to work engagement (0.05, *p* = 0.25) were non-significant. All hypothesized reversed causal paths were significant, except for the path linking identified regulation to workaholism (0.09, *p* = 0.06). The path coefficients for workaholism impacting on introjected regulation and intrinsic motivation were 0.12 (*p* = 0.01) and −0.08 (*p* = 0.02), respectively, whereas the path coefficients for work engagement impacting on identified regulation and intrinsic motivation were 0.13 (*p* = 0.01) and 0.41 (*p* < 0.001), respectively. The results of the reciprocal model agree almost completely with the combined results of the causality and reversed causal models; only the paths from T1 intrinsic motivation to T2 work engagement and from T1 identified regulation to T2 workaholism were non-significant. Although the fit of the reciprocal model was comparable to that of the reversed causality model (M2_*reversed causality*_), Δχ^2^(*N* = 314, *df* = 5) = 8.52, *n.s.*, the reversed causality model was preferred because it was more parsimonious. After removing non-significant paths from this model, the model (M2_*final*_) still fitted the data well, χ^2^(*N* = 314, *df* = 71) = 179.97, GFI = 0.94, CFI = 0.97, NFI = 0.95, TLI = 0.95, RMSEA = 0.070. Figure 3 presents the final model. Again, the correlations between the error terms of the indicator variables of the latent variables in this figure (Time 1 and Time 2 work engagement) were omitted for clarity.

Regarding the associations between *motivation and workaholism*, Hypotheses 2a and 2b asserted that introjected regulation and identified regulation would have a positive effect on workaholism, respectively. Figure 3 shows that workaholism at Time 1 predicted introjected regulation at Time 2 instead of the other way around (β = 0.12, Hypothesis 2a rejected). We found no significant relations between identified regulation and workaholism (Hypothesis 2b rejected). Furthermore, whereas Hypothesis 2c stated that intrinsic motivation would have a negative effect on workaholism, a reversed negative effect of workaholism at Time 1 on intrinsic motivation at Time 2 was obtained (β = −0.09, Hypothesis 2c rejected).

As for the associations between *motivation and work engagement*, Hypotheses 3a and 3b proposed that intrinsic motivation and identified regulation would affect work engagement positively. We found significant associations between these two kinds of motivation and work engagement. However, as with workaholism, these associations were in the reversed direction: work engagement at Time 1 predicted intrinsic motivation (β = 0.41) and identified regulation (β = 0.12) at Time 2 (Hypotheses 3a and 3b rejected).

### *Post-hoc* Analyses

#### Need Satisfaction and Heavy Work Investment

Cole and Maxwell (2003) argue that mediation processes in two-wave models should be examined by focusing on two separate models, one examining the associations between the presumed “antecedents” and the “mediators” and the other examining the associations between the “mediators” and the “outcomes” (cf. Figure 1). However, it is also of interest to examine the associations between the “antecedents” (in this case, need satisfaction) and the “outcomes” (engagement and workaholism). Therefore, we conducted an additional series of structural equation analyses, comparing a stability model, a causality model, a reversed causality model and a reciprocal model for the longitudinal associations among need satisfaction and both types of heavy work investment. The stability model, involving only lagged effects of Time 1 need satisfaction and the two types of heavy work investment on the same variables as measured at Time 2, fitted the data best, χ^2^(*N* = 314, *df* = 64) = 189.54, GFI = 0.93, CFI = 0.96, NFI = 0.95, TLI = 0.95, RMSEA = 0.079. The other three models yielded fit values that were identical to those obtained for the stability model (for GFI, CFI, NFI, and TLI) or that were slightly worse. Specifically, RMSEA decreased for these models, varying from 0.80 to 0.082, and their χ^2^-values varied from 185.61 to 186.70: since these three models were slightly more complex than the stability model they possessed fewer degrees of freedom, meaning that the decreases in their χ^2^-values were not statistically significant (all *p*s for these chi-square difference tests were >0.24). These findings suggest that need satisfaction is not an antecedent of engagement or workaholism.

#### Motivation and Heavy Work Investment

Broadly speaking, different types of motivation were expected to affect workaholism and work engagement across time. However, Figure 3 shows that – contrary to our hypotheses – both need satisfaction and heavy work investment affect motivation over time. To examine the unique contributions of these predictors, an additional model with cross-lagged effects of need satisfaction and the two types of heavy work investment at Time 1 on the different types of motivation at Time 2 was examined, controlling for the stability of these different types of motivation. This model fitted the data well, χ^2^(*N* = 314, *df* = 157) = 391.75, GFI = 0.90, CFI = 0.95, NFI = 0.92, TLI = 0.93, RMSEA = 0.069, showing that workaholism at Time 1 predicted introjected regulation at Time 2 (β = 0.11), and work engagement at Time 1 predicted identified regulation (β = 0.12) and intrinsic motivation (β = 0.38) at Time 2. Thus, workaholism and work engagement as measured at T1 accounted for a significant part of the variance in at least some of the motivational variables at T2, even after controlling for Time 1 need satisfaction, and the stabilities of the motivational variables.

## Discussion

The present study is among the first to study work motivation and heavy work investment longitudinally. Drawing on Deci and Ryan (2000) self-determination theory (SDT), this study examined how need satisfaction affects work motivation and how work motivation affects workaholism and work engagement across time. Although our findings are partly in line with previous theorizing and research (Deci et al., 2017) the present study also calls into question some prior beliefs.

First, the current study showed that need satisfaction forestalls external regulation and introjected regulation, but promotes intrinsic motivation across time. The extent to which the needs for autonomy, competence, and relatedness are satisfied seems to have no effect on identified regulation. These findings might suggest that employees who struggle with unsatisfied needs become more motivated by threats of punishments or material and social rewards (external regulation), and by partially internalized external standards of self-worth and social approval (introjected regulation). They experience a desire to be in control, to master their environment, and to feel connected with others (Deci and Ryan, 2000) and their work compensates unmet needs (Mageau et al., 2009). As a consequence, they are hindered in acting in line with their personal values and interests (Deci et al., 2017). Since external standards and partially adopted external standards might conflict with what employees personally prefer, they might feel pressured to work (Ryan and Deci, 2000). This is in line with Bartolomew et al. (2011) observation that athletes’ psychological need thwarting was related to higher levels of burnout, depression and negative affect. In contrast, employees with fulfilled needs are able do what they find interesting and enjoyable (intrinsic motivation). They will engage in their work for its own sake with a full sense of volition (Deci and Vansteenkiste, 2004). This type of behavior embodies the growth-oriented tendency of human beings, and as a result these employees will flourish. Therefore, the present findings underline the necessity of fulfilled innate psychological needs for optimal work motivation.

Second, the present study unexpectedly showed that workaholism promotes introjected regulation and reduces intrinsic motivation across time, rather than that these types of motivation regulation predicted workaholism. Apparently, workaholic employees become more motivated by partially internalized external standards of self-worth and social approval (introjected regulation). They are driven to demonstrate their competencies and to avoid failure in order to achieve feelings of self-worth, like pride, and to avoid feelings of shame, guilt, and worthlessness (Ryan and Deci, 2000). Since this type of motivation is accompanied by an internal pressure to behave in particular ways, employees will be hindered in pursuing goals that fit their genuine ideals and interests (Ryan et al., 1991). In other words, their intrinsic motivation and, thus, their growth-oriented nature will be undermined. Consistent with this reasoning, the present study revealed a negative effect of workaholism on intrinsic motivation, suggesting that over time, employees who work excessively and compulsively will find their work less interesting and enjoyable than others, possibly due to the depletion of resources resulting from high effort expenditure.

Third, the present study showed that work engagement leads to identified regulation and intrinsic motivation across time. Engaged employees become more motivated by the underlying value of their work (identified regulation), and the pleasure and satisfaction that they derive from their work (intrinsic motivation). Like workaholic employees, engaged employees may become more extrinsically motivated across time. While workaholics may adopt external standards of self-worth and social approval which in turn regulate their motivation (introjection), engaged employees will recognize the underlying value of their work and will more fully internalize external standards (identification). The external standards seem to become part of their identity (Deci et al., 2017) and as a result, they experience ownership of their behavior (Ryan and Deci, 2000). In addition, they will do their work because they find the work activities attractive. Therefore, it is likely that engaged employees’ growth-oriented nature can take its own course and that engaged employees will flourish (Deci and Vansteenkiste, 2004).

Finally, the findings presented here did not support our reasoning that the associations between need satisfaction and heavy work investment (i.e., workaholism and work engagement) are mediated by motivation (cf. Figure 1). Although the expected effects of need satisfaction on motivation were largely supported (step 1 in Figure 1), workaholism and work engagement predicted motivation rather than that they were predicted by motivation (step 2 in Figure 1). Since our post-hoc analyses showed that need satisfaction and heavy work investment were unrelated longitudinally, it appears that heavy work investment (engagement and workaholism) are predictors of motivation, next to need satisfaction. One possible interpretation draws on Schneider’s Attraction-Selection-Attrition framework, stating that workers are attracted to and will actively look for jobs that fit their personality, attitudes, interests, values and capacities (Schneider et al., 2000) and that good fit between the job and the worker will result in positive outcomes such as high intrinsic motivation. Thus, engaged workers will be attracted to jobs providing good possibilities for identified and intrinsic motivation and workaholics will seek for jobs that offer opportunities for experiencing identified and introjected, rather than intrinsic motivation. This reasoning implies that workaholism and work engagement can be considered as relatively stable personal characteristics. There is indeed some evidence that major personality factors and heavy work investment are related (Burke et al., 2006; Inceoglu and Warr, 2011) suggesting that these two forms of heavy work investment have at least some temporally stable component. Furthermore, workaholism and work engagement may be associated with job crafting behavior. For example, engaged workers are likely to increase the resources present in their jobs (Makikangas, 2018) which may in turn lead to more opportunities for experiencing intrinsic motivation. Interesting as these notions may be, at present they remain largely speculative and require more research before they can be accepted as a reasonable interpretation of the findings presented here.

### Study Limitations

Four main limitations of the present study must be discussed. First, this study is based on a convenience sample, and therefore we have only modest insight in the type of employees that participated in our study. As participants were recruited through a call on an internet site addressing career-related issues, our participants may well have been more interested in career-related information than the average Dutch employee, e.g., because they were looking for a new job or because they explored the possibilities for further development (in a material and/or developmental sense) in their present job. This might have led to a restriction of the range of the scores on the study concepts, lack of statistical power, and conservatively estimated effect sizes.

Second, the present study relied exclusively on self-report data. Using a single source might have exaggerated the associations between our study variables due to common method variance. However, Spector and Brannick (2009) convincingly show that self-report studies do not necessarily lead to inflated correlations and that the role of social desirability is often overestimated. Furthermore, the strength of the associations displayed in Table 2 varies considerably, suggesting that the associations have not been influenced by a common underlying process that affects all these associations uniformly. This was confirmed by the confirmatory factor analyses presented in Table 1, showing that at neither of the study waves a single latent factor accounted well for the associations among the observed variables. One possible option for mitigating the issue of self-report bias would be to include objective data. Although this would not seem feasible for the present set of study variables as these all concern intra-individual evaluations of own motivation, engagement and work drive, future research could extend these concepts with measures of objective performance, e.g., as rated by colleagues of supervisors.

Third, the present study revealed small cross-lagged effects. It might be that our study design, a relatively basic two-wave design with a 6-month interval, has undermined these effects (Hakanen et al., 2008). Specifically, one unresolved methodological issue in longitudinal research concerns the optimal length of the time intervals between the study waves: time intervals that are too short may lead to the conclusion that no causal effects exist due to the absence of any meaningful change in the study variables, whereas time intervals that are too long may lead to an underestimation of the causal effects due to the fact that participants may change “back and forth” regarding their scores on the study variables (Taris, 2000; Ford et al., 2014). It is sometimes argued that short intervals (of less than 1 year or even 6 months) usually correspond better with the “true” causal interval than longer intervals (Dormann and Griffin, 2015). Others argue that stressor-strain effects are strongest for a time lag of 2–3 years, and that this “optimal” length varies strongly for different types of variables (Ford et al., 2014). Moreover, no similar study has been conducted for the concepts in the present study. In this sense, previous research provides no strong guidelines regarding the optimal time lag between the sstudy variables for the present set of variables. Consequently, in selecting a time lag for the present study we felt that a 6-month interval between the study waves presented a reasonable compromise between “short” and “long” intervals: a shorter period would not be sufficient for employee well-being to be affected by need satisfaction via motivational regulation, while effects might have vanished after a longer period.

Furthermore, the length of the right time lag will also depend on the temporal stability of the study variables. In the present study we controlled for stability effects. Due to the relatively stable nature of our study concepts (with Time 1-Time 2 correlations being in the range of 0.50–0.70, cf. Table 2), the predictor variables might have been unable to explain much variance in the outcomes variables (Taris and Kompier, 2006). The baseline level of a concept at Time 1 was the most important predictor of the scores on the same concept at Time 2. However, our model fitted the data acceptably well and revealed several significant cross-lagged effects, suggesting that the 6-month interval was a reasonable approximation of the “true” time lag of at least some of the processes studied here.

Therefore, it would be interesting for future research to replicate our findings using *multiple* waves with follow-ups at, for instance, 6, 12, and 18 months. This would allow for testing the mediation effect of motivational regulation on the study outcomes as a function of the time lag used in the study. It would also maximize the chances of including the “right” time lag for studying the association between need satisfaction and employee well-being (Taris and Kompier, 2016). As such this would contribute to more insight into the underlying dynamics of heavy work investment. However, given the relatively high Time 1-Time 2 nonresponse rate of 81.4%, we felt that the effort in conducting a third wave would exceed the possible benefits of including a third wave, as we considerd it likely that this would result in a unusably low sample size.

A related venue for future research would be to follow newcomers who just started their jobs. This would circumvent the current problem of providing only a Time 1-Time 2 snapshot, while employees will usually have experienced certain levels of need satisfaction, motivation and wellbeing before Time 1 as well. Using a baseline score that is collected right at the start of their jobs, for instance among school-leavers, would give us more insight in the initial unfolding of the process.

Further, in examining the associations among need satisfaction, motivation and heavy work investment this study focused on the associations with need satisfaction as an overall (latent) concept, rather than on the three separate indicators of this concept (i.e., autonomy satisfaction, competence satisfaction and relatedness satisfaction). In line with earlier findings (Van den Broeck et al., 2010) in our study these three types of need satisfaction could empirically be considered as indicators of a single overarching latent construct at both study waves. However, the patterns of associations between these three types of need satisfaction and other variables may vary across type of need satisfaction (Van den Broeck et al., 2016). Although at the measurement level these needs were taken as separate indicators of the latent construct Need satisfaction (which allows for differential effects of these needs on the study outcomes through their factor loadings on the Need satisfaction concept), this procedure focuses on what the three types of need satisfaction have in common, rather than on their possibly different associations with other variables. The correlation coefficients presented in Table 2 show that whereas the *direction* of the associations between the three types of need satisfaction and other study variables was the same for all types of need satisfaction, their *magnitude* varied to a certain extent as a function of type of need satisfaction. Although our structural equation analyses demonstrated that these differences were not large enough to invalidate our findings, it may still be worthwhile for future research to examine the associations between need satisfaction and other variables as a function of type of need satisfaction.

Finally, the present study sought to examine the associations among need satisfaction, different types of motivation, and two forms of heavy work investment (engagement and workaholism). It did *not* aim to disentangle their interrelationships or their underlying dynamics. That is, interesting and possibly important questions regarding their relative importance in affecting other concepts, their conceptual and empirical distinction, or possible transitions from one type of heavy work investment to another, were beyond this study. However, the present data set presents some information relevant to these issues. For example, the correlations presented in Table 1 show that the test-retest correlations for (indicators of) workaholism and engagement are in the range of 0.70 and higher. Apparently, these concepts tend to be rather stable during the 6-month interval observed here, which suggests that although transitions will have occured, their number would probably be small and not necessarily meaningful. Thus, research aiming to study the pattern of transitions from workaholism to engagement and vice versa would probably need to cover at least a year. Moreover, it is interesting to consider the associations between workaholism and engagement, specifically, conceptually it would be reasonable to expect workaholics to be “absorbed” by their jobs. This should be reflected in relatively high, positive associations between especially the absorption indicator of engagement and the workaholism concept. Previous research on this issue provided mixed evidence for this idea, with Schaufeli et al. (2009) reporting low, negative associations beween engagement and workaholism (of −0.05 and −0.19 for Japan and Netherlands, respectively) and no cross-loadings of the indicators of these concepts. However, in a similar study Schaufeli et al. (2008) found that the absorption indicator of engagement also loaded substantially on workaholism (a standardized loading of 0.39). The present study found that the correlations between absorption and workaholism ranged from 0.03 to 0.14, speaking against the notion that absorption is an important part of workaholism. Clearly, there is a need for future research to examine the associations between these two forms of heavy work investment in more detail. *Study implications*. Despite these limitations, the present study advances our knowledge in several ways. First, the present study supports SDT’s assumption that the extent to which the three innate psychological needs are fulfilled determines employees’ motivation (Deci et al., 2017). Hence, need satisfaction represents an essential psychological process through which external standards are internalized and integrated, and intrinsic goal pursuit is facilitated (Deci and Vansteenkiste, 2004). To enhance need satisfaction managers may create an optimal work environment. Specifically, in order to support employees’ need for autonomy, managers may clarify the purpose of work activities when assigning these tasks to them (Van den Broeck et al., 2008). Also, managers may offer employees choices and give employees the opportunity to make decisions (Van den Broeck et al., 2009). To support their need for competence, managers may offer employees challenging activities and training, and provide them with positive feedback (Van den Broeck et al., 2008). Lastly, to support the need for relatedness managers may encourage close relationships at work by regular meetings and organizing lunch breaks centrally.

Second, the current study raises some questions about SDT’s assumption that different regulatory processes underlying goal pursuits make an important difference in terms of effective functioning at work; i.e., heavy work investment (Deci and Ryan, 2000). Specifically, the current study challenges the assumed causal order in SDT in which motivational regulation precedes heavy work investment. However, it should be noted that a slightly less parsimonious model (M2_*reciprocal*_) also fitted the data well and showed that motivation and the two types of heavy work investment reciprocally affect each other (Stoeber et al., 2013). Nevertheless, based on the preferred model, workaholism and work engagement both affect work motivation, but in different ways. This finding also suggests that it would be appropriate to consider workaholism and work engagement as two different phenomena that predispose employees to act in certain ways. Workaholism seems to promote employees’ inclination to engage into self-protective behavior, a process marking the experience of negative emotions (Lyubomirsky et al., 2005). As regards the origin of workaholism, it may seem equivalent to a specific set of personal characteristics, like perfectionism, a strong need for achievement, and compulsiveness (Mudrack, 2004). Also, workaholism may result from and maintained by distorted cognitions (McMillan et al., 2003). For example, it is suggested that workaholic employees are insecure and have a negative self-view (Mudrack, 2006; Shimazu et al., 2019). Based on the present findings, workaholic’s behavior becomes motivated by (partially) internalized external standards of social approval and self-worth. Meeting these standards results in feelings of high self-worth and self-esteem, whereas failing to meet these standards leads to self-criticism and negative affect (Ryan and Deci, 2002).

Work engagement seems to predispose employees to pursue self-concordant goals, a process marking the experience of positive emotions (Judge et al., 2005; Lyubomirsky et al., 2005). Favorable work environments (e.g., autonomy, social support from colleagues and supervisors, and performance feedback) and personal resources (e.g., self-efficacy and optimism) foster the development of work engagement (Bakker and Demerouti, 2008). A favorable work environment stimulates employees to do their very best, and increases the chance that work tasks are successfully completed and work goals are successfully achieved. The conviction that one is capable to reach goals and that good things will happen also contributes to positive outcomes. Employees who can draw upon these personal resources are ideally suited to take advantage of opportunities to broaden and build their repertoire of skills (Lyubomirsky et al., 2005). Free from negative feelings and distress, they can actively pursue goals that they value and find inherently satisfying (Judge et al., 2005).

Workaholism and work engagement may also lead employees to drift to certain jobs (Zapf et al., 1996). For instance, in selection, employees with high levels of social competence, self-esteem, and stress tolerance are preferred for skilled jobs. As a result, engaged employees may get the better jobs, i.e., the jobs that allow them to do what they find important (identified regulation), and enjoyable and interesting (intrinsic motivation). Furthermore, it might be that engaged employees’ high energy levels and positive beliefs stimulate them to search for jobs that they value and find inherently enjoyable and interesting.

### Concluding Comment

The present study is among the first to provide longitudinal evidence on the processes that underlie work motivation. Although we did not find the expected effects of motivation on the two types of heavy work investment in this study, the present study supported the important role of need satisfaction for motivation and challenged theoretically plausible ideas on the effects of motivation on workaholism and work engagement. Although more – especially longitudinal – research is needed regarding the latter issue, workaholism can certainly be considered a bad type of heavy work investment and work engagement a good type of heavy work investment.

## Author’s Note

This manuscript has been released as a Pre-Print at www.schaufeli.com (Van Beek, 2014).

## Data Availability Statement

The datasets generated for this study are available on request to the corresponding author.

## Ethics Statement

This study was conducted in accordance with the guidelines of the Declaration of Helsinki. Participants were informed about the goal of the study before actually participating. Participation was voluntary and anonymous and participants could withdraw from the study whenever they wanted. No particularly sensitive topics were involved, participants were not subjected to deception and completed a non-invasive questionnaire. According to our university’s research policy, this sort of research is exempted from approval from our faculty’s Ethical Research Committee.

## Author Contributions

TT, IB, and WS were involved in designing the study and writing up its results. IB collected the data and analyzed the results, with an additional contribution by TT. All authors contributed to the article and approved the submitted version.

## Conflict of Interest

The authors declare that the research was conducted in the absence of any commercial or financial relationships that could be construed as a potential conflict of interest.
